# Effect of Dietary Westernization on Inflammatory Bowel Disease in Japan

**DOI:** 10.31662/jmaj.2021-0148

**Published:** 2021-09-21

**Authors:** Kiichiro Tsuchiya

**Affiliations:** 1 Department of Gastroenterology, Faculty of Medicine, University of Tsukuba, Ibaraki, Japan

**Keywords:** dietary westernization, FODMAP, emulsifier

The increase in the number of patients with inflammatory bowel disease (IBD) in Asia is remarkable. Therefore, it is important to investigate its cause to suppress the increase. The pathogenic factors of IBD are complex and are divided mainly into genetic and environmental factors. In the West, the genetic factor is believed to carry a higher weight mainly because of the identification of disease susceptibility genes for IBD and their functional analysis. However, Asian patients with IBD do not have the disease susceptibility genes possessed by Western patients, suggesting that the increase in the number of patients in Asia is influenced largely by environmental factors ^(1)^. In this issue, Chiba et al. focused on dietary westernization among various environmental factors in Japan. They showed that dietary westernization and the increase in patients with IBD were concurrently promoted over time ^(2)^. It is of great significance that this study, using a national survey, revealed what many Japanese doctors had envisioned. However, as the author indicated, it is only a chronological match without any relevance. Further examination is required for the elucidation of a correlation between dietary westernization and the onset of IBD. Basic studies have already reported that emulsifiers transform the gut microbiota and thin the mucous layer of the intestinal mucosa ^(3)^. As these emulsifiers are present in ice cream, instant noodles, mayonnaise, pastry, chocolate, etc., the intake of emulsifiers by Japanese people might be increasing because of dietary westernization. In addition, reports on the effects of a high-fat diet on intestinal immunity are increasing. Therefore, it is expected that inflammation will be induced by an excessive immune response. Persistent inflammation has also been reported to acquire the irreversible fragility of intestinal epithelial cells. Normal human colonic epithelial cells with inflammatory stimulation for over a year, resulted in the suppression of cell proliferation even after the inflammatory stimulation was stopped. Moreover, IBD-like crypt formation, such as goblet cell depletion and crypt distortion, was shown ^(4)^. Taken together, a series of steps - diet, gut bacteria dysbiosis, immune control disruption, chronic inflammation, and mucosal fragility - are thought to complete a pathogenic form of IBD. Although genetic factors also increase the risk of developing IBD, environmental factors might mainly contribute to the onset of IBD and the increase in the number of patients with IBD, especially in Asia.

Dietary westernization is useful information from the viewpoint of not only the onset factor of IBD but also IBD treatment. In recent years, a FODMAP diet has been the focus of interest of researchers. FODMAP is an acronym for Fermentable, Oligosaccharides, Disaccharides, Monosaccharides, and Polyols. Diets may be divided into high-FODMAP diets (apples, pears, asparagus, broccoli, wheat, pasta, cookies, milk, yogurt, sorbitol, nuts, etc.) and low-FODMAP diets (bananas, strawberries, grapefruits, carrots, eggplants, potatoes, pumpkins, rice, tofu, sugar, etc.). Although a high-FODMAP diet, which is part of Western diets, is said to disturb the intestinal environment, a low-FODMAP diet has been reported to be effective in alleviating abdominal symptoms of patients with IBD ^(5)^. Further studies on FODMAP diets for IBD treatment are necessary in the future.

Another fact is that meals are taken every day. It is difficult to continue taking an ideal diet throughout one’s life. Diet is restricted not only by personal preference but also by economic reasons and the time taken to prepare meals. It is also necessary to consider diet from a social aspect, such as the overflow of cheap and quick foods, such as fast foods and instant foods. Although patients and doctors talk about the necessity of a healthy diet, it is difficult to spread this information to all the people in a country. It seems that it will take time to change the capital economy for the small number of patients compared to the population in Japan.

Now that we have discussed the relationship between food and IBD, we need to consider other factors that change over time. For example, in Japan, the infection rate of *Helicobacter pylori* is decreasing during the same decades. This is thought to be due to changes of hygiene in the environment, regardless of the westernization of the diet. The decrease in the infection rate of *H. pylori* is also associated with an increase in gastric acid, which has a considerable effect on the intestinal microbiota.

In conclusion, it is important to extract all environmental factors without biasing the idea only to diet and intestinal microbiota. A comprehensive evaluation about environmental factors will lead to a reduction in the future onset of IBD and improve its therapeutic options.

## Article Information

### Conflicts of Interest

None
